# Building Workforce Capacity to Deliver Effective Behavioral Health Support for Older Adults with Hoarding & Clutter

**DOI:** 10.1093/geroni/igaf122.1632

**Published:** 2025-12-31

**Authors:** Bronwyn Keefe, Jordana Muroff, Annalee Wilson, Crystal Carrington, Megan Nizza

**Affiliations:** Boston University School of Social Work, Boston, Massachusetts, United States; Boston University, Boston, Massachusetts, United States; Boston University, Boston, Massachusetts, United States; Boston University, Boston, Massachusetts, United States; Boston University, Boston, Massachusetts, United States

## Abstract

The goal of this project was to develop and implement capacity-wide training and programming focused on hoarding and clutter to build behavioral health supports and services to address this growing concern. This collaboration between a private University and an Area Agency on Aging (AAA) in a large urban area focused on addressing the urgent and growing priority area of hoarding and clutter among culturally and linguistically diverse older adults. The training aimed to enhance the understanding of hoarding as a mental health challenge, teach evidence-based assessments and intervention strategies, and apply effective communication and engagement techniques and community approaches. We delivered and evaluated a blended hoarding training (online and in-person) to a broad group of AAA providers (n = 130) working with individuals with hoarding, e.g., case managers, nurses, management staff, caregiver advisers, and direct care staff. The blended training includes an innovative online interactive self-paced course and “live” practice-based training sessions delivered by hoarding experts. Before and after the training program, learners self-reported their skill level on nine competencies related to working with individuals with a hoarding disorder. Learners showed statistically significant (p < 0.05) improvements in all competencies. Learners also rated their confidence (How confident do you feel working with people with hoarding?) and comfort (Do you feel comfortable with clients with hoarding?) before and after the training program. Upon completing the in-person training session, learners were significantly (p < 0.05) more confident and comfortable working with clients with hoarding disorder. These findings highlight the impact of providing high-quality training and effective hoarding-related interventions.

